# A Case Report of the Largest Submucosal Uterine Leiomyoma Removed by Single-Session Hysteroscopic Myomectomy in the Caribbean

**DOI:** 10.7759/cureus.22985

**Published:** 2022-03-09

**Authors:** Vishal Bahall, Lance De Barry, Keevan Singh, Narika Singh

**Affiliations:** 1 Obstetrics and Gynaecology, The University of the West Indies, St. Augustine, TTO; 2 Obstetrics and Gynaecology, South-West Regional Health Authority, San Fernando, TTO; 3 Obstetrics and Gynaecology, San Fernando General Hospital, San Fernando, TTO; 4 Anaesthesia and Intensive Care Unit, Department of Clinical Surgical Sciences, The University of the West Indies, San Fernando, TTO; 5 Internal Medicine, South-West Regional Health Authority, San Fernando, TTO

**Keywords:** fertility, hysteroscopy, myomectomy, fibroids, submucous leiomyoma

## Abstract

Minimally invasive gynecological surgery is rapidly evolving in the Caribbean. Hysteroscopic myomectomy is the procedure of choice for the removal of submucosal uterine leiomyomas. In Trinidad and Tobago, advancements in minimally invasive surgery have allowed submucosal myomectomies to be performed hysteroscopically with results that are on par with international standards of care. This report highlights a successful hysteroscopic myomectomy performed for the largest submucosal uterine leiomyoma documented in the Caribbean.

## Introduction

Uterine leiomyomas are benign tumors of the myometrium that are present in up to 80% of women in the reproductive age group in the Caribbean [1]. It is the most common indication for myomectomy and hysterectomy in the region [1]. Although most women are asymptomatic, uterine leiomyomas are associated with significant morbidity and symptomatic women often report menorrhagia, dysmenorrhoea, pelvic pain disorders, infertility, or early pregnancy complications [2].

The mainstay of fertility-sparing surgical treatment for leiomyomata is uterine myomectomy [1]. Traditionally, the abdominal approach to myomectomy has been the standard of care in the Caribbean for intracavitary uterine leiomyomas. Internationally, hysteroscopy is the gold standard modality used for evaluating the uterine cavity and treating submucosal leiomyomas [3]. Perioperative outcomes with hysteroscopic myomectomy have been shown to be superior when compared to abdominal myomectomy with uterine cavity exploration for submucosal leiomyomas [4]. Hysteroscopic myomectomy is associated with lower postsurgical morbidity, improved fertility, faster rates of recovery, shorter duration of hospitalization, and an overall safer surgical experience for patients [4].

Trinidad and Tobago, a twin-island developing state in the Caribbean, have been at the forefront of minimally invasive gynecological surgical techniques in the region and has been keeping up with international advancements in gynecological surgery. In this report, we highlight the case of a successful submucosal myomectomy, and, to the best of our knowledge after an extensive literature review, the removal of the largest submucosal leiomyoma done by hysteroscopy in the Caribbean.

## Case presentation

A 39-year-old nulliparous female presented to the Gynaecology clinic with menorrhagia, dysmenorrhea, and symptomatic anemia for one year. She denied experiencing weight loss, fever, and urinary or gastrointestinal symptoms. She had a history of symptomatic uterine leiomyoma that was treated with an abdominal myomectomy five years earlier. She subsequently had menorrhagia and dysmenorrhea after three years. An ultrasound at this time revealed a submucosal fibroid measuring 6.1 cm in diameter. Before her visit to our clinic, the patient used different medical treatment options such as goserelin (3.6 mg subcutaneously once per month for six months), medroxyprogesterone acetate, and tranexamic acid with minimal improvement over one year. Her medical history was otherwise unremarkable.

**Figure 1:**
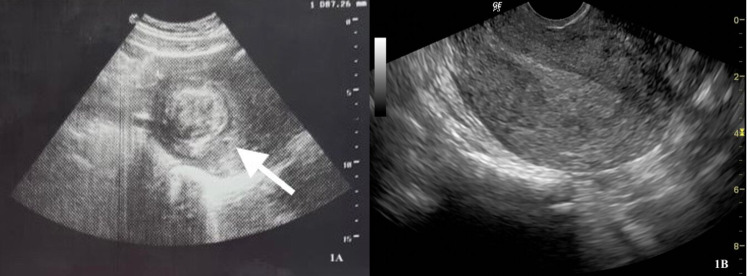
Pelvic ultrasound scan of the uterus. (A) Preoperative scan demonstrating a 6.9 cm × 3.6 cm submucosal leiomyoma (white arrow). (B) Postoperative scan demonstrating complete removal of the intracavitary leiomyoma.

On clinical examination, a 10-week-sized firm uterus was palpated in the pelvis. Speculum examination demonstrated a healthy-appearing cervix. Blood investigations revealed microcytic anemia (hemoglobin 8.8 g/dL, mean corpuscular volume 73 fL) and normal renal and liver function tests. Pelvic ultrasonography revealed an enlarged uterus with a 6.9 cm × 3.6 cm solitary mass within the endometrial cavity (Figure 1, Panel A). The mass was postulated to be a large submucosal leiomyoma. The patient was counseled on fertility-sparing and non-fertility-sparing surgical treatment options. She desired fertility-sparing minimally invasive surgery and opted for a hysteroscopic myomectomy.

**Figure 2:**
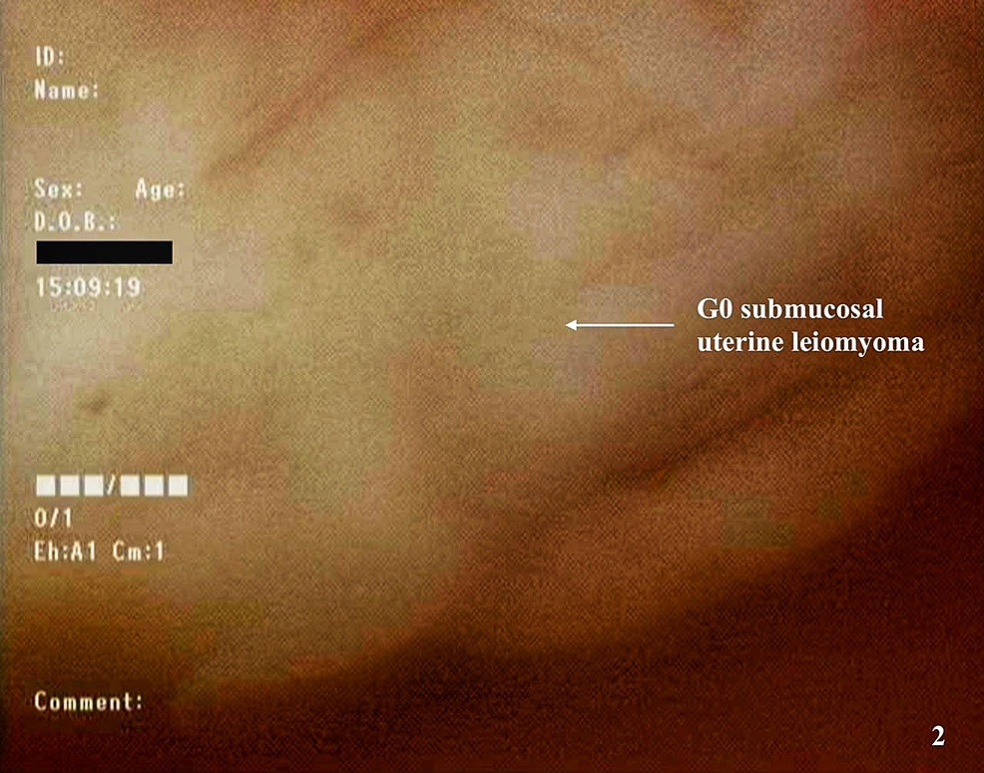
Hysteroscopic view of the large G0 submucosal uterine leiomyoma.

Intraoperatively, the uterine cavity was entered using a 10 mm 0° hysteroscope following sufficient cervical dilatation under general anesthesia. The uterine cavity was distended with 0.9% saline solution at a pressure of 100 mmHg facilitated by the Thermedx FluidSmart^TM^ (Thermedx, Solon, OH, USA) fluid management system. A panoramic inspection of the uterine cavity was first performed to assess the intracavitary topography, and a solitary submucosal G0 leiomyoma (completely endocavitary leiomyoma with no intramural extension) was observed on the right lateral wall of the uterine cavity which was consistent with preoperative imaging (Figure 2). The TruClear^TM^ Ultra 8 mm hysteroscopic morcellator (Smith and Nephew, Andover, MA, USA) was inserted into the endocervical canal under direct visualization. The leiomyoma was resected down to its base under direct visualization, and the morcellated tissue fragments were suctioned through the device into a collecting pouch for postoperative histopathological analysis (Video 1). The total operative duration was 145 minutes, and the estimated blood loss was 50 mL. The total volume of distending medium used was approximately 10 L of 0.9% saline solution, and the fluid deficit was approximately 1,200 mL. The patient exhibited no signs of fluid overload.

**Video 1:** Morcellation of the submucosal leiomyoma using the TruClearTM ultra 8mm hysteroscopic morcellator.

The patient’s postoperative course was unremarkable, and she was discharged within 24 hours of the procedure. At follow-up visits 10 days and six weeks later, the patient was well with no complaints. She reported resolution of her heavy vaginal bleeding, and postoperative pelvic ultrasonography demonstrated no remnants of the submucosal leiomyoma (Figure 1, Panel B).

## Discussion

The incidence of submucosal leiomyomas of the uterus is approximately 5-10% [5]. Although less common than subserosal and intramural leiomyomas, submucosal myomas are associated with greater morbidity, including dysmenorrhea, menorrhagia, symptomatic anemia requiring blood transfusions, infertility, and an increased risk of early pregnancy complications [5]. Depending on the severity of symptoms, treatment options may be medical or surgical. Medical management includes hormonal therapy such as oral contraceptives, single-agent progesterone suppression, or gonadotrophin-releasing hormone agonists [6]. Hormonal treatment is often combined with non-hormonal options that include non-steroidal anti-inflammatory drugs and tranexamic acid [6]. While the aim of medical management is to control menorrhagia, these agents do not improve fertility and rarely impact the size of leiomyomas. In this regard, surgical management becomes necessary [2].

Hysteroscopic myomectomy is currently the gold standard fertility-sparing treatment modality for submucosal uterine leiomyomas [3]. Traditionally, abdominal myomectomy represented the only possibility of preserving fertility in young patients afflicted by symptomatic submucosal leiomyomas [3]. The abdominal approach to intracavitary leiomyomas can, in some instances, reduce rather than enhance fertility secondary to the development of postoperative intrauterine adhesion formation and can compromise future parturition because patients often require a cesarean section [4]. Hysteroscopic myomectomy provides superior perioperative outcomes and improved fertility rates compared to abdominal myomectomy for submucosal leiomyomas [4]. Advancements in minimally invasive gynecological procedures and training in the Caribbean have allowed hysteroscopy to become a practical approach to myomectomy and improve the morbidity of women undergoing leiomyoma surgery in the region [7].

Hysteroscopic myomectomy can be a technically challenging, complex procedure and requires appropriate preoperative evaluation to determine the feasibility of the procedure. To minimize incomplete resection and procedural complications, patients should undergo preoperative transvaginal ultrasonography, saline infusion sonography, or outpatient hysteroscopy and biopsy to evaluate the leiomyoma characteristics [2]. Alternatively, magnetic resonance imaging can accurately delineate uterine anatomy, as well as the size, number, and location of uterine leiomyomas; however, its cost and availability may limit its routine use [8]. In addition, patients susceptible to complications of fluid overload should undergo appropriate preoperative cardiovascular evaluation and risk assessment [9].

Based on saline infusion sonography and outpatient hysteroscopy, submucosal leiomyomas can be classified according to the criteria developed by Wamsteker et al. and later adopted by the European Society of Gynaecological Endoscopy (ESGE) (Table 1) [10]. Large leiomyomas with greater myometrial penetration are associated with decreased operative success rates, increased surgical risks, or the requirement of additional surgical sessions to complete resection. Mazzon et al., in a large cohort study of single-step hysteroscopic myomectomy involving 1,244 patients, concluded that all G0 myomas can be removed in a single surgical procedure regardless of myoma size, whereas G2 leiomyomas greater than 3 cm, as opposed to G1 leiomyomas, are correlated with a higher risk of multiple procedures [11]. Therefore, most gynecologists prefer the hysteroscopic approach to myomectomy for submucosal leiomyomas with a diameter of 3 cm or less [11].

**Table 1:** European Society of Gynaecological Endoscopy classification of submucosal leiomyomas.

Type of submucosal leiomyoma	Characteristics
G0	Entirely within the endometrial cavity with no myometrial extension
G1	<50% myometrial extension, <90-degree angle of leiomyoma surface to the uterine wall
G2	>50% myometrial extension, >90-degree angle of leiomyoma surface to the uterine wall

Several techniques have been developed to effectively achieve complete hysteroscopic resection of G0 and G1 leiomyomas. The two major modalities available to facilitate leiomyoma removal include the hysteroscopic tissue removal system (TruClear^TM^ hysteroscopic morcellator) and hysteroscopic resectoscopes [4]. Several notable advantages exist regarding the use of hysteroscopic morcellation over conventional resectoscopes. According to retrospective comparisons, hysteroscopic morcellation is associated with shorter procedure times and shorter learning curves, is easier to use, provides better intraoperative visibility because it easily collects and removes tissue fragments for later analysis, and has a better safety profile because it reduces the need for additional instrumentation within the uterine cavity [3,12]. In comparison, hysteroscopic resectoscopes are associated with longer operating times, a steeper learning curve, requires greater skill to utilize, and may require additional surgical sessions to complete leiomyoma resection [12]. Regardless of the technique, the aim is to reduce injury to the underlying myometrium by converting the intramural myoma into a complete intracavitary lesion [3]. In some cases, incomplete removal of intracavitary myomas may not require subsequent additional surgery as uterine contraction typically occurs with removal of G1 leiomyomas, and retained fragments are often expelled vaginally [4]. Notwithstanding, patients should be advised on the possibility of incomplete resection and the need for additional surgical procedures.

This operation was completed by a one-step hysteroscopic procedure that achieved complete excision of the large G0 6.9 cm × 3.6 cm leiomyoma. To our knowledge, after an extensive literature review, this is the largest submucosal leiomyoma removed by hysteroscopic myomectomy in the Caribbean. The largest submucosal leiomyoma managed by hysteroscopic myomectomy in the Caribbean measured less than 4 cm in diameter [7]. The lead surgeon, a highly trained minimally invasive gynecologist, completed the procedure in accordance with the standard technique for hysteroscopic myomectomy. The utilization of the TruClear^TM^ hysteroscopic morcellator and Thermedx FluidSmart^TM^ fluid management system allowed for the seamless disintegration, removal, and collection of the myoma specimen for later histopathological analysis. The total operative time in our case was 145 minutes compared to the international average of 105 minutes [13]. One factor that led to the extended operative duration, in this case, was the large size and surface area of the leiomyoma.

Additionally, an intrauterine pressure between 70 mmHg and 100 mmHg facilitated using distending medium is required for adequate visualization of the uterine cavity [14]. As noted in our case, larger leiomyomas may demand an extended operating duration. Therefore, to avoid excessive absorption of the distending medium, the intrauterine pressure should be kept below the mean arterial pressure, and the fluid deficit should be monitored closely [15]. Fluid deficits are more accurately monitored in a closed system where the fluid is actively suctioned and returned to a reservoir, as done by the Thermedx FluidSmart^TM^ fluid management system [14]. According to the ESGE guidelines, a maximum fluid deficit of 2,500 mL should be set when using an isotonic solution (such as 0.9% saline solution) in a healthy woman, and the procedure should be immediately terminated upon reaching this limit to avoid complications of fluid overload [14]. In our case, the fluid deficit was approximately 1,200 mL, and the patient did not exhibit any signs of fluid overload.

## Conclusions

The field of minimally invasive gynecology is rapidly advancing in the Caribbean with rising popularity among both gynecologists and patients. Complex hysteroscopic interventional procedures should be performed by trained surgeons to maintain proper quality control and minimize adverse procedural outcomes. Hysteroscopic resection of submucosal leiomyomas improves fertility, menorrhagia, symptomatic anemia, and reduces early pregnancy complications. This case highlights the largest submucosal leiomyoma removed by single-session hysteroscopic myomectomy in the Caribbean as a day case.
